# Pathway analysis of rare variants for the clustered phenotypes by using hierarchical structured components analysis

**DOI:** 10.1186/s12920-019-0517-4

**Published:** 2019-07-11

**Authors:** Sungyoung Lee, Sunmee Kim, Yongkang Kim, Bermseok Oh, Heungsun Hwang, Taesung Park

**Affiliations:** 1 Center for Precision Medicine, Seoul National University Hospital, Seoul, Korea; 2 Department of Psychology, McGill University, Montreal, Canada; 3 Department of Statistics, Seoul National University, Seoul, Korea; 4 Department of Biochemistry and Molecular Biology, School of Medicine, Kyung Hee University, Seoul, Korea; 5 Interdisciplinary Program in Bioinformatics, Seoul National University, Seoul, Korea

**Keywords:** Generalized estimating equations, Clustered phenotypes, Pathway analysis, Rare variants

## Abstract

**Backgrounds:**

Recent large-scale genetic studies often involve clustered phenotypes such as repeated measurements. Compared to a series of univariate analyses of single phenotypes, an analysis of clustered phenotypes can be useful for substantially increasing statistical power to detect more genetic associations. Moreover, for the analysis of rare variants, incorporation of biological information can boost weak effects of the rare variants.

**Results:**

Through simulation studies, we showed that the proposed method outperforms other method currently available for pathway-level analysis of clustered phenotypes. Moreover, a real data analysis using a large-scale whole exome sequencing dataset of 995 samples with metabolic syndrome-related phenotypes successfully identified the glyoxylate and dicarboxylate metabolism pathway that could not be identified by the univariate analyses of single phenotypes and other existing method.

**Conclusion:**

In this paper, we introduced a novel pathway-level association test by combining hierarchical structured components analysis and penalized generalized estimating equations. The proposed method analyzes all pathways in a single unified model while considering their correlations. C/C++ implementation of PHARAOH-GEE is publicly available at http://statgen.snu.ac.kr/software/pharaoh-gee/.

## Backgrounds

The history of Genome-Wide Association Studies (GWAS) now has reached two decades, and those GWAS have identified almost 60,000 unique associations of over 3000 traits [1]. However, despite the steeply increasing GWAS discoveries, those discoveries explain only a small portion of expected phenotypic variations [2, 3], a phenomenon known as “missing heritability” [2]. Some of the possible explanation for such phenomenon include gene-gene interaction, pleiotropic effect, and rare variants [3].

For the analysis of rare variants, the low statistical power caused by the sparseness of rare variants is one of the major issues. The use of biological information such as genes or pathways has been proven to escalate the statistical power and improve the biological interpretation, for identifying statistically significant genes and pathways associated with complex traits such as high-density lipoprotein levels, obesity, schizophrenia, and multiple cancers [4–8]. Taking the advantages of the pathway-level analysis, we have developed statistical methods PHARAOH that investigates pathway-level associations [9] and PHARAOH-multi that extends PHARAOH to the analysis of multiple continuous phenotypes [10]. Our PHARAOH method has two exclusive features. First, it employs the hierarchy of biological process by constructing a hierarchical structural model of the rare variants, genes, pathways, and phenotype(s). Second, it considers all pathways within a single unified model with statistical regularization, hence effectively controlling the correlations between genes and pathways.

Another approach to improving the statistical power is a simultaneous analysis of clustered phenotypes. For example, the analysis of repeatedly measured phenotypes outperforms the analysis of cross-sectionally observed phenotypes, since the information on the temporal differences within a subject improves the power [11]. Many recent GWAS have analyzed the repeatedly measured phenotypes and discovered many novel associations, such as fasting glucose, body mass index, and lung function [12–14]. In the repeated measures analysis, a consideration of the correlations between the repeated measurements is crucial. Neglecting the nature of clustered phenotypes may result in loss of statistical power [15].

The Generalized Estimating Equations (GEE) approach is one of the most commonly used methods for the analysis of clustered and correlated phenotypes [15]. The major advantages of GEE include that it can handle a wide class of phenotypes such as binary, count, and continuous traits from an exponential family distribution and that its estimator is consistent regardless of the specification of the working correlation structure. In these respects, the GEE approach has been contributed to the discovery of genetic components from various studies including association studies of lung cancer [16], ophthalmological measurements [16, 17], and gene-drug interaction analysis [18]. For the analysis of expression datasets, various extensions of GEE have been proposed such as the repeated microarray experiment and penalized GEE for microRNA dataset [17, 18]. For gene-level tests, several GEE methods have been developed, including Longitudinal Genetic Random Field (LGRF) and GEE-KM [19, 20].

However, unlike the gene-level analyses, to the best of our knowledge, only one method based on GEE has employed the pathway-level analysis of the correlated phenotypes [21] with the R package GEEaSPU. Note that GEEaSPU employs the adaptive Sum of Powered score (aSPU) and adapts the GEE framework to enable pathway-level analysis of genetic variants [21]. However, the GEEaSPU method cannot handle the correlations between the pathways, which can result in the biased results.

In order to address this problem, we propose a novel pathway-level association test for clustered and correlated phenotypes such as repeated measurements, Pathway-based approach using HierArchical component of collapsed RAre variants Of High-throughput sequencing data using Generalized Estimating Equations (PHARAOH-GEE). While the existing GEE based pathway-level method GEEaSPU implements the individual “pathway-wise” test assuming all tests are independent, the proposed PHARAOH-GEE method implements a “global test” that considers the correlation among the pathways into account by putting all pathways simultaneously into a single model. Moreover, PHARAOH-GEE can handle various types of phenotypes (e.g., binary), and it also retains the advantages of PHARAOH, such as the hierarchical model that mimics the natural biological processes. By providing PHARAOH-GEE program using a powerful and fast C/C++ based framework WISARD [22], it supports various genetic data formats and provides affordable performance.

## Results

We used a workstation system consists of two Intel Xeon E5–2640 CPUs and 256GiB of RAM. Due to the limitation of the compared method, the R version 3.4.0 and R package ‘GEEaSPU’ were used with default settings.

### Simulation study

For our simulation study, we generated 300 replicates from the simulated data pool. Each replicate consisted of 10 pathways in which the first pathway was causal and the other nine were non-causal (i.e., no effect). For each replicate, the proposed PHARAOH-GEE method was applied to the 10 pathways simultaneously, whereas GEEaSPU was applied to each pathway individually. Here we assumed that the first pathway is causal and the others are non-causal. For the causal pathway, we considered three different parameter settings: four gene-level effects (*w =* 0.1, 0.2, 0.5 and 1.0), three pathway-level effects (*β* =0.15, 0.2 and 0.25), two correlations of phenotypes (*ρ* =0.25 and 0.5). For all test results, we applied the BH step-up procedure to control the False Discovery Rate (FDR) at 5% level [23]. Details on simulation procedure can be found on Methods section.

**Fig. 1:**
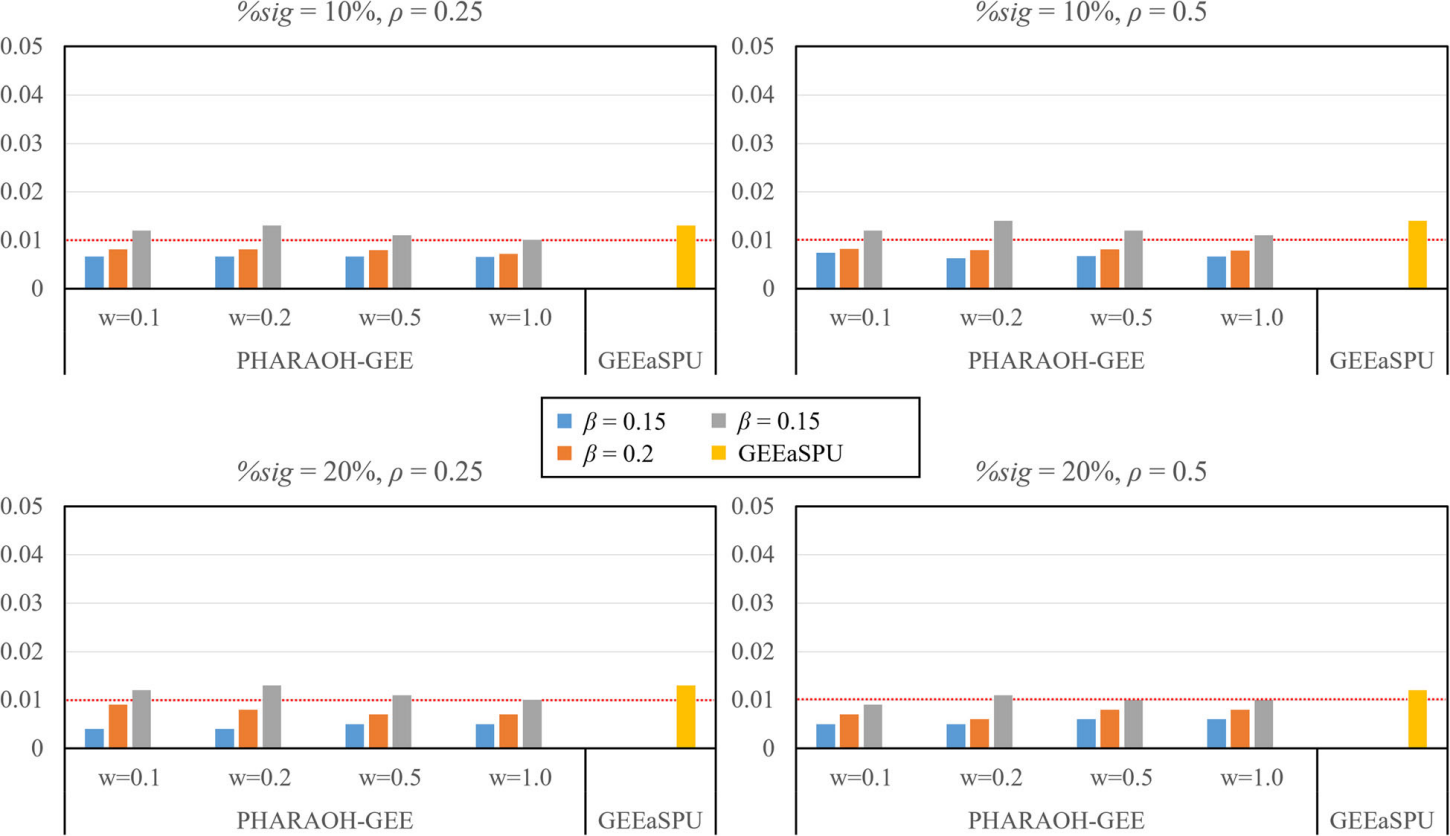
Results of type 1 error simulation. Rows represent the proportions of significant genes within the causal pathway (10 and 20%), and columns represent different phenotypic correlation (0.25 and 0.5). For each plot, type 1 errors of PHARAOH-GEE are shown with varying gene-level effects (0.1, 0.2, 0.5 and 1.0) and pathway-level effects (0.15, 0.2 and 0.25), and type 1 errors of GEEaSPU are shown with orange bars

First, we evaluated the type 1 errors of PHARAOH-GEE and GEEaSPU. For the given parameter settings for the causal pathway, we evaluated the type 1 errors using 9 non-causal pathways with significance level *α* = 0.01. As shown in Fig. 1, all methods controlled the type 1 error rates appropriately, regardless of the parameter values.

**Fig. 2:**
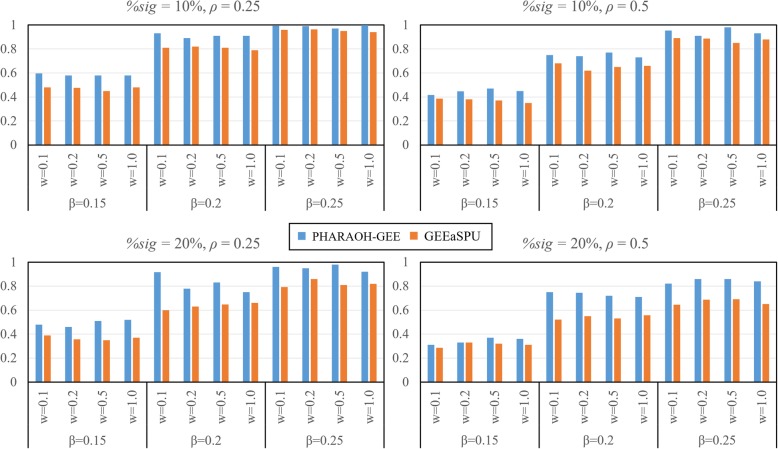
Result of power analysis. Columns and rows represent different phenotypic correlations (0.25 and 0.5) and proportions of significant genes within the causal pathway (10 and 20%). For each plot, estimated statistical powers from 300 simulation datasets are shown with combinations of gene-level effects (0.1, 0.2, 0.5 and 1) and pathway-level effects (0.15, 0.2 and 0.25)

Second, we evaluated statistical power of the methods where power was computed as a proportion of the causal pathway being statistically significant at the FDR < 0.05 over 300 replicates. In addition to three parameter settings for the causal pathway, we consider two cases when the numbers of significant genes within the causal pathway are only one (*H*_1_ = 1) and two (*H*_1_ = 2) out of ten simulated genes, respectively. As shown in Fig. 2, PHARAOH-GEE outperforms GEEaSPU in all simulation scenarios.

In the power analysis, there were two additional interesting findings. First, when the proportion of significant genes in the causal pathway became smaller, the proposed method tended to outperform GEEaSPU. Second, PHARAOH-GEE showed less reduction of statistical power than GEEaSPU when the phenotypic correlation *ρ* increased. In real practical situation where only a fraction of genes is likely related to phenotypes and that the correlations among clustered phenotypes are high, these findings suggest that PHARAOH-GEE would be more powerful for detecting true biological signals than GEEaSPU.

### Analysis of whole exome sequencing (WES) dataset using clustered phenotypes

To demonstrate the usefulness of PHARAOH-GEE, we analyzed a large-scale sequencing dataset with six phenotypes related to the metabolic syndrome: systolic blood pressure (SBP), diastolic blood pressure (DBP), triglycerides (TG), fasting glucose (FASTGLU), waist circumference (WAIST), and high-density lipoprotein (HDL). Before the analysis, we binarized these phenotypes according to the metabolic syndrome criteria of International Diabetes Federation (IDF) consensus worldwide definition of the metabolic syndrome (https://www.idf.org). Metabolic syndrome is diagnosed as the presence of three or more of the following criteria: (1) WAIST ≥90 cm in males and ≥ 80 cm in females; (2) elevated TG ≥ 150 mg/dL or taking medication; (3) HDL-cholesterol < 40 mg/dL in males and < 50 mg/dL in females or taking lipid-lowering agents; (4) systolic blood pressure ≥ 130 mmHg or diastolic blood pressure ≥ 85 mmHg or taking antihypertensive medications; and (5) elevated FASTGLU ≥100 mg/dL or oral hypoglycemic agents use. From these six metabolic syndrome related phenotypes, we derived five clustered binary traits. Especially, we combined two blood pressure phenotypes (SBP & DBP) into a single phenotype, named BP, by setting 1 if either SBP or DBP satisfied the diagnosis criteria of metabolic syndrome and 0 otherwise. All other phenotypes were binarized if the diagnosis criteria of metabolic syndrome was satisfied and 0 otherwise.

**Fig. 3:**
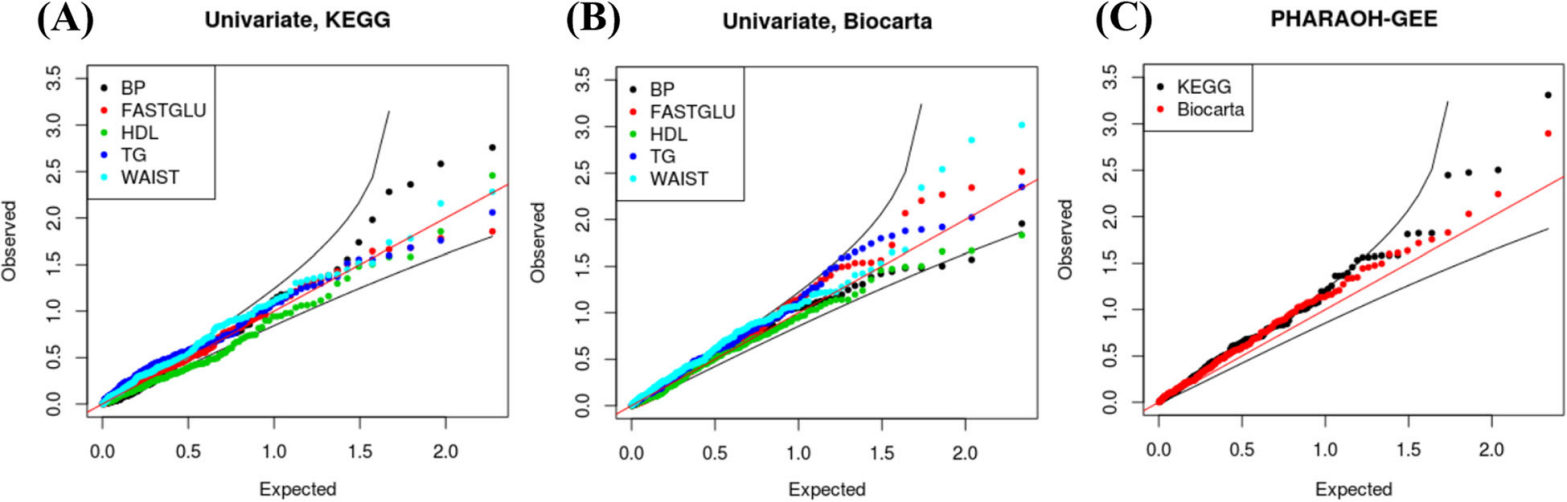
Q-Q plots of the real data analyses. **a** Q-Q plot of the univariate analyses using KEGG pathway database and **b** Biocarta pathway database. **c** Q-Q plot of the analysis of the five phenotypes using PHARAOH-GEE

We applied PHARAOH for the univariate analysis of each binary phenotype and applied PHARAOH-GEE and GEEaSPU for the multivariate analysis of the five binary phenotypes. We conducted the multiple testing adjustment to both univariate and multivariate analyses by using the BH step-up procedure [23]. The unstructured covariance structure of the phenotypes was assumed for both PHARAOH-GEE and GEEaSPU. Figure 3 presents quantile-quantile (Q-Q) plots showing that PHARAOH and PHARAOH-GEE led to no substantial deflation or inflation of *p*-values.

Table 1 exhibits the pathways with the five smallest q-values identified by PHARAOH-GEE, as well as their q-values under PHAROH and GEEaSPU. PHARAOH-GEE was able to identify one KEGG pathway, the glyoxylate and dicarboxylate metabolism, at the *q*-value threshold of 0.1. None of these pathways turned out to be statistically significant in the univariate analyses of PHARAOH, always resulting in larger *q*-values than those from PHARAOH-GEE. Although the same glyoxylate and dicarboxylate pathway had the lowest *p*-value by GEEaSPU, it failed to pass the *q*-value threshold of 0.1, after the multiple testing adjustment. Thus, our real data analyses showed the relatively superior performance of PHARAOH-GEE.

**Table 1:** Top five pathways from PHARAOH-GEE. The *q*-values after the multiple testing adjustment are presented in each cell, with their corresponding *p*-values within the brackets. The results of univariate PHARAOH are also provided on the right side of the table

Pathway	PHARAOH-GEE	GEEaSPU	Univariate PHARAOH				
			HDL	TG	FASTGLU	WAIST	BP
Glyoxylate and dicarboxylate metabolism	0.0929 (0.00063)	0.16 (0.00099)	0.987 (0.902)	0.721 (0.021)	0.772 (0.023)	0.91 (0.842)	0.916 (0.202)
Glycosphingolipid biosynthesis ganglio series	0.159 (0.0038)	0.979 (0.804)	0.987 (0.79)	0.805 (0.658)	0.855 (0.137)	0.805 (0.359)	0.695 (0.067)
MAPK signaling pathway	0.159 (0.00404)	0.468 (0.126)	0.987 (0.327)	0.721 (0.234)	0.855 (0.072)	0.953 (0.901)	0.997 (0.45)
Valine-leucine and isoleucine biosynthesis	0.159 (0.0043)	0.979 (0.797)	0.987 (0.242)	0.871 (0.779)	0.999 (0.801)	0.91 (0.813)	0.695 (0.067)
Fatty acid metabolism	0.436 (0.0173)	0.977 (0.459)	0.987 (0.834)	0.721 (0.143)	0.999 (0.893)	0.903 (0.647)	0.997 (0.909)

Among the five pathways identified by PHARAOH-GEE, a recent study suggests a strong relationship between the metabolic syndrome and two pathways (glyoxylate and dicarboxylate, and fatty acid metabolisms), through their role in abdominal obesity [24]. In addition, the glycosphingolipid biosynthesis and MAPK signaling pathways are reported to be related to the metabolic syndrome via insulin resistance that plays a critical role in manifestation of the metabolic syndrome [25, 26].

## Conclusion

An analysis of the clustered phenotypes provides more information than the cross-sectional studies. Recent large cohort studies keep producing repeatedly measured phenotypes. We introduced a novel statistical method for the pathway analysis of the large-scale genetic dataset with clustered phenotypes. While our previous PHARAOH-multi method can handle only continuous phenotypes, the proposed PHARAOH-GEE can handle various phenotypes such as clustered binary and count phenotypes under the various correlation structures. Through the comparison study using the simulated datasets, we demonstrated that the proposed PHARAOH-GEE method outperforms an existing pathway method. Furthermore, our application to the large-scale WES dataset successfully identified one pathway that has not been discovered in the analyses of individual phenotype with the multiple testing adjustments.

## Discussion

Compared to GEEaSPU the only currently available method for pathway-level test of clustered phenotypes, the proposed method has many advantages. First, PHARAOH-GEE effectively controls the complex correlations among the pathways by constructing a unified hierarchical, doubly-penalized statistical model. Second, it successfully reflects the nature of biological process from GSCA framework and takes clustered phenotypes into account from GEE framework. In conclusion, we hope that PHARAOH-GEE can serve as a main tool for the pathway-level analysis of clustered phenotypes in genetic studies.

Currently, we have a number of considerations for our future research. Although we considered many possible combinations of parameters in the simulation setting, a further extensive simulation study is required for more comprehensive comparison with existing pathway-based methods. In addition, we will perform a replication study using other independent datasets with the metabolic syndrome phenotypes. Finally, we will employ other penalization methods such as lasso and elastic-net.

## Methods

### PHARAOH-GEE method

**Fig. 4:**
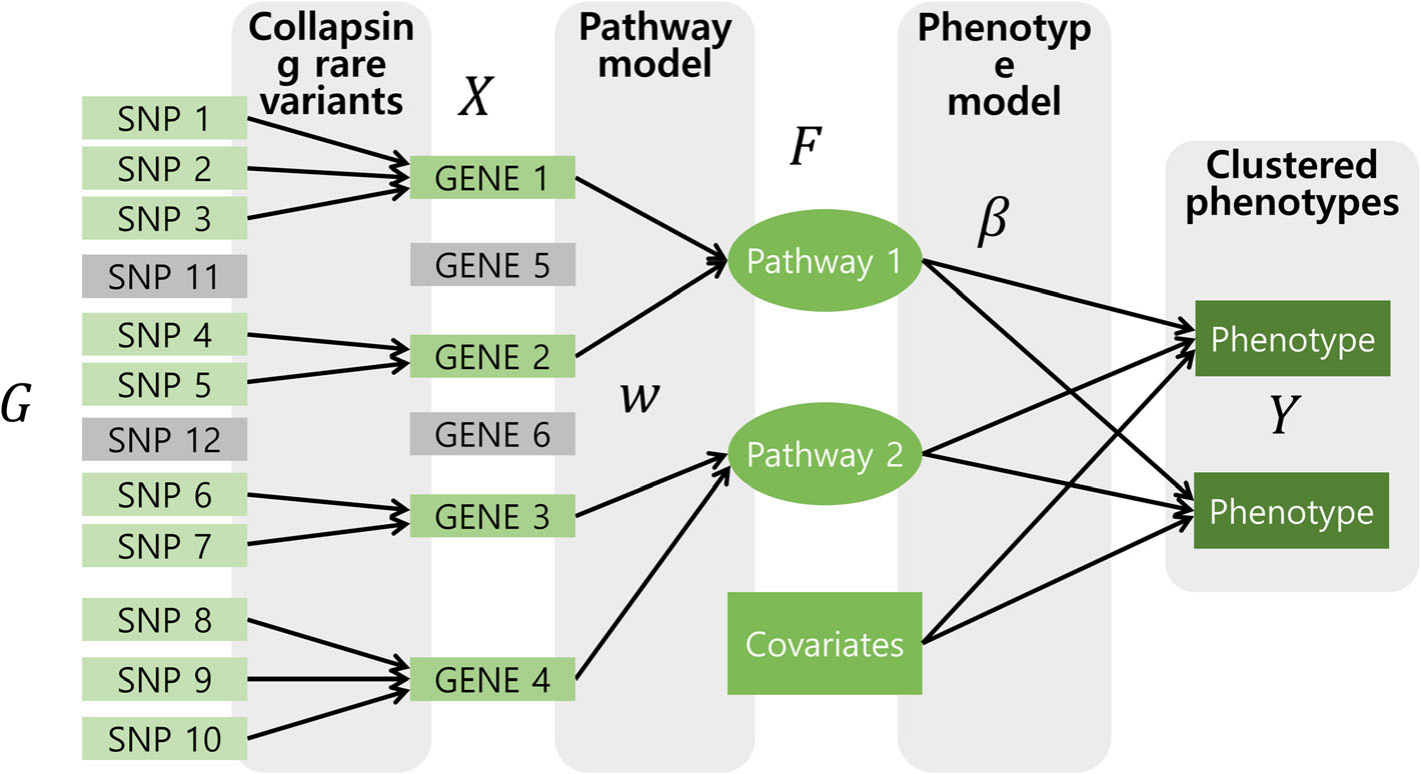
An example of the PHARAOH-GEE model

Technically, the proposed method is an extension of the doubly-regularized Generalized Structured Component Analysis into the GEE framework [27] that imposes ridge penalties [28] on both gene-pathway and pathway-phenotype relationships. From the previous studies, we successfully demonstrated that those two ridge penalties effectively control the correlations between genes and pathways [9, 10]. PHARAOH-GEE aims to identify associations between *Q* clustered phenotypes and *K* pathways, each of which is linked to *T*_*k*_ genes (*k* = 1, ⋯, *K*). An example of the PHARAOH-GEE model is depicted in Fig. 4.

Let *y*_*iq*_ be the value of the *q*^th^ phenotype measured on the *i*^th^ individual (*i* = 1, …, *N*; *q* = 1, …, *Q*) and $\tilde{y}_{i}={\left[{y}_{i1},\cdots, {y}_{iQ}\right]}^{\prime }$ be a *Q* × 1 vector of the clustered phenotypes of the *i*^th^ individual. Similar to the previous description of the PHARAOH model [9], we assume that *y*_*iq*_ follows an exponential family distribution with a mean *μ*_*iq*_. Let Σ_*i*_ be the *Q* × *Q* covariance matrix of $\tilde{y}_{i}$. Then,

1 $$ \operatorname{cov}\left(\tilde{y}_{i}\right)={\Sigma}_{i\ \left(Q\times Q\right)}={A}_i^{1/2}{R}_i\left(\alpha \right){A}_i^{1/2}, $$

where *R*_*i*_(*α*) is a so-called “working correlation matrix”, *α* is a parameter vector that fully characterizes *R*_*i*_(*α*), and ${A}_i^{1/2}=\operatorname{diag}\left[\operatorname{var}\left({\mu}_{ij}\right)\right]$, i.e., a *Q* × *Q* diagonal matrix with the marginal variance of responses. Liang and Zeger [29] suggested various choices for *R*_*i*_(*α*), e.g., the independence covariance structure, *R*_*i*_(*α*) = **I**_*Q*_, where **I**_*Q*_ is the identity matrix of order *Q*.

Let $\tilde{x}_{i}^{\prime }=\left[1,\cdots, 1;{x}_{i11},\cdots, {x}_{i1{T}_1};\mathbf{\cdots};{x}_{iK1},\cdots, {x}_{iK{T}_K}\right]$ be a (*T* + 1) × 1 vector consisting of all gene-level collapsed variables for the *i*^th^ individual across *K* pathways, where *T* = ${\Sigma}_{k=1}^K{T}_k$. The gene-level collapsed variables are generated as the weighted sums of rare variants. Let **X** be an *N* × (*T* + 1) matrix of the gene-level collapsed variables for *N* observations, as expressed in (2).

2 $$ {\mathbf{X}}_{N\times \left(T+1\right)}=\left[\ \begin{array}{ccccc}1& {x}_{111}& {x}_{112}& \cdots & {x}_{1K{T}_K}\\ {}1& {x}_{211}& {x}_{212}& \cdots & {x}_{2K{T}_K}\\ {}\vdots & \vdots & \vdots & \ddots & \vdots \\ {}1& {x}_{N11}& {x}_{N12}& \cdots & {x}_{NK{T}_K}\end{array}\right]=\left[\begin{array}{c}{\overset{\sim }{x}}_1^{\prime}\\ {}{\overset{\sim }{x}}_2^{\prime}\\ {}\vdots \\ {}{\overset{\sim }{x}}_N^{\prime}\end{array}\right]. $$

As in the previous methods [9], we standardize **X** to satisfy the conventional scaling constraint diag(**X**^′^**X**) = *N***I**. Each element of **X**, *x*_*ikt*_, denotes a gene-level summary of the *i*^th^ sample for the *t*^th^ gene (*t* = 1, ⋯, *T*_*k*_) in the *k*^th^ pathway and is generated by the weighted sum of rare variants that is same as the previous work [9, 10]. Let *W* denote a (*T* + 1) × (*K* + 1) matrix consisting of component weights *w*_*tk*_, which are assigned to *x*_*ikt*_. This matrix can be generally expressed as

3 $$ {\boldsymbol{W}}_{\left(T+1\right)\times \left(\mathrm{K}+1\right)}=\left[\ \begin{array}{ccccc}1& 0& 0& \cdots & 0\\ {}0& {w}_{11}& 0& \cdots & 0\\ {}\vdots & \vdots & \vdots & \ddots & \vdots \\ {}0& {w}_{1{T}_1}& 0& \cdots & 0\\ {}0& 0& {w}_{21}& \cdots & 0\\ {}\vdots & \vdots & \vdots & \ddots & \vdots \\ {}0& 0& {w}_{2{T}_2}& \cdots & 0\\ {}\vdots & \vdots & \vdots & \vdots & \vdots \\ {}0& 0& 0& \cdots & {w}_{K1}\\ {}\vdots & \vdots & \vdots & \ddots & \vdots \\ {}0& 0& 0& \cdots & {w}_{K{T}_K}\end{array}\right]. $$

Let *η*_*iq*_ and *g*(·) denote the *i*^th^ linear predictors of the *q*^th^ phenotype and a link function, respectively. We define the proposed PHARAOH-GEE model as

4 $$ g\left({\mu}_{iq}\right)={\eta}_{iq}={\beta}_{0q}+\sum \limits_{k=1}^K\left(\sum \limits_{t=1}^{T_k}{x}_{ik t}{w}_{tk}\right){\beta}_{kq}={\beta}_{0q}+\sum \limits_{k=1}^K{f}_{ik}{\beta}_{kq}={\overset{\sim }{\boldsymbol{f}}}_i{\overset{\sim }{\boldsymbol{\beta}}}_q, $$

where ${f}_{ik}={\sum}_{t=1}^{T_k}{x}_{ik t}{w}_{tk}$ is the component score of the *i*^th^ individual for the *k*^th^ pathway ${\overset{\sim }{\boldsymbol{f}}}_i=\left[1,{f}_{i1},\cdots, {f}_{iK}\right]$, and ${\overset{\sim }{\boldsymbol{\beta}}}_q=\left[{\beta}_{0q}\ {\beta}_{1q}\cdots {\beta}_{Kq}\right]$ is a vector of coefficients linking *K* pathways to the *q*^th^ phenotype. We can statistically examine the joint effects of the *k*^th^ pathway on *Q* phenotypes by testing the null hypothesis H_0_: *β*_*k*1_ = ... = *β*_*kQ*_ = 0. Moreover, it is possible to evaluate the effect of one gene on a single phenotype mediated by its corresponding pathway.

### Parameter estimation

For simplicity, we describe the propose method, assuming that the phenotype $\tilde{y}_{i}$ is continuous. It is technically straightforward to extend the method to other phenotypes from exponential distributions. In parameter estimation, we add two *L*_2_ penalty terms to control for potential adverse influences of high correlations between genes and/or pathways. Specifically, to estimate the parameters ***W*** and ***B***, we seek to minimize the following penalized estimating equations.

5 $$ {\phi}_{\alpha, B,W}=\sum \limits_{i=1}^N{U}_i+{\lambda}_P tr\left({\boldsymbol{B}}^{\prime}\boldsymbol{B}\right)+{\lambda}_G tr\left({\boldsymbol{W}}^{\prime}\boldsymbol{W}\right), $$

where *U* is the estimating equation for the parameters, ***B*** is a matrix consisting of all regression coefficients ${\overset{\sim }{\beta}}_q$, *tr*(·) denotes the trace of matrix, and *λ*_*G*_ and *λ*_*P*_ denote ridge parameters on the *L*_2_ penalty terms for the weights and regression coefficients, respectively. A more detail on the estimating equation and solving process can be found on elsewhere [9].

To minimize *ϕ*_*α*, *B*, *W*_, we use an iterative algorithm that repeats the following steps until no substantial changes in parameter estimates occur.

**Step 1**: We update ***B*** for fixed ***W*** and *R*_*i*_(*α*). Let ***b*** **=** vec(***B***) denote a vector formed by stacking all columns of *B* one below another. This is equivalent to minimizing the following estimating equations

6 $$ {\phi}_1=\sum \limits_{i=1}^NU\left(\boldsymbol{b}\right)+{\lambda}_P{\boldsymbol{b}}^{\prime}\boldsymbol{b}=\sum \limits_{i=1}^N\ \left({\boldsymbol{f}}_i^{\prime}\otimes \mathbf{I}\right){\Sigma}_i^{-1}\left({y}_i-\left({\boldsymbol{f}}_i^{\prime}\otimes \mathbf{I}\right)\boldsymbol{b}\right)+{\lambda}_P{\boldsymbol{b}}^{\prime}\boldsymbol{b}=\sum \limits_{i=1}^N{\boldsymbol{Q}}_i{\Sigma}_i^{-1}\left({y}_i-{\boldsymbol{Q}}_i\boldsymbol{b}\right)+{\lambda}_P{\boldsymbol{b}}^{\prime}\boldsymbol{b}, $$

where ${\boldsymbol{Q}}_i={\boldsymbol{f}}_i^{\prime}\otimes \mathbf{I}$ and ⊗ denotes Kronecker product. Then, ***b*** can be estimated by $\hat{\boldsymbol{b}}={\left(\sum \limits_{i=1}^N{\boldsymbol{Q}}_i^{\prime }{\Sigma}_i^{-1}{\boldsymbol{Q}}_i+{\lambda}_P\mathrm{I}\right)}^{-1}\left(\sum \limits_{i=1}^N{\boldsymbol{Q}}_i^{\prime }{\Sigma}_i^{-1}{y}_i\right)$, and $\hat{\boldsymbol{B}}$ is reconstructed from $\hat{\boldsymbol{b}}$.

**Step 2**: We update ***W*** for fixed ***B*** and *R*_*i*_(*α*). Let ***w*** **=** vec(***W***). Similar to step 1, it is equivalent to minimizing

7 $$ {\phi}_2=\sum \limits_{i=1}^NU\left(\boldsymbol{w}\right)+{\lambda}_G\boldsymbol{w}^{\prime}\boldsymbol{w}=\sum \limits_{i=1}^N{\left(\tilde{x}_{i}^{\prime}\otimes {B}^{\prime}\right)}^{\prime }{\Sigma}_i^{-1}\left({y}_i-\left(\tilde{x}_{i}^{\prime}\otimes {B}^{\prime}\right)w\right)+{\lambda}_G{w}^{\prime }w=\sum \limits_{i=1}^N{M}_i^{\prime }{\Sigma}_i^{-1}\left({y}_i-{M}_i{w}_{\ast}\right)+{\lambda}_G{w}_{\ast}^{\prime }{w}_{\ast }, $$

where ${M}_i=\tilde{x}_{i}^{\prime}\otimes {\boldsymbol{B}}^{\prime },$ w* is the vector formed by eliminating all zero elements of ***w***, and *M*_*i*_ is the matrix formed by removing the columns of $\tilde{x}_{i}^{\prime}\otimes {B}^{\prime }$ corresponding to the zero elements of w. Then, *w*_∗_ can be estimated by ${\hat{\boldsymbol{w}}}_{\ast }={\left(\sum \limits_{i=1}^N{M}_i^{\prime }{\Sigma}_i^{-1}{M}_i+{\lambda}_G\mathrm{I}\right)}^{-1}\left(\sum \limits_{i=1}^N{M}_i^{\prime }{\Sigma}_i^{-1}{z}_i\right).$ Then, the estimated ***W*** is reconstructed from ${\hat{\boldsymbol{w}}}_{\ast }$.

**Step 3**: We update *R*_*i*_(*α*) from the updated ***B*** and ***W*** using Pearson residuals with the variance function of the distribution *ν*,

8 $$ {r}_{ij}=\left({y}_{ij}-{\hat{\mu}}_{ij}\right)/{\nu}^{1/2}\left({\hat{\mu}}_{ij}\right). $$

where ${\hat{\mu}}_{ij}={\beta}_{0q}+\sum \limits_{k=1}^K{f}_{ik}{\hat{\beta}}_{kq}.$ Finally, the dispersion parameter *φ* is estimated consistently by

9 $$ \hat{\varphi}={\left( NQ-\left(K+\sum \limits_{k=1}^K{T}_k\right)\right)}^{-1}\sum \limits_{i=1}^N\sum \limits_{j=1}^Q{\hat{r}}_{ij}^2. $$

We apply *k*-fold cross-validation (CV) to estimate the values of *λ*_*G*_ and *λ*_*P*_, which compares the quasi-deviance values [30] of a two-dimensional grid of candidate values of *λ*_*G*_ and *λ*_*P*_.

### Significance testing and multiple correction

Resampling methods can be used to test the statistical significance of the estimated effects of all pathways on a given set of clustered phenotypes. In the proposed method, we utilize a permutation test to obtain *p*-values. By permuting the phenotypes, the method first generates the empirical null distributions of both pathways and gene coefficients. By computing the quantile of the estimated pathway and gene coefficients from the non-permuted dataset with the corresponding null distribution, we can obtain an empirical *p*-value for any specific pathway and gene.

In our study, we want to test the joint effects of pathways on clustered phenotypes. In our previous study, we introduced two approaches to test *β*_*k*1_, ..., *β*_*kQ*_ simultaneously and suggested the Wald-type statistics [10]. Similarly, we construct a single statistic that combines all *Q* coefficients. Here, we define a Wald-type statistic *T* as.

10 $$ T={\overset{\sim }{\boldsymbol{\beta}}}_k^{\prime }{\operatorname{cov}}^{-1}\left({\overset{\sim }{\boldsymbol{\beta}}}_k\right){\overset{\sim }{\boldsymbol{\beta}}}_k. $$

Under penalized GEE, the estimated covariance $\operatorname{cov}\left({\hat{\overset{\sim }{\beta}}}_k\right)$ can be obtained in two ways. One way is to calculate it directly, as introduced by Wang et al. [31] as follows.

11 $$ \operatorname{cov}\left(\hat{\overset{\sim }{\boldsymbol{\beta}}}\right)={\left({H}_{\hat{\overset{\sim }{\beta }}}+n{E}_{\hat{\overset{\sim }{\beta }}}\right)}^{-1}{M}_{\hat{\overset{\sim }{\beta }}}{\left({H}_{\hat{\overset{\sim }{\beta }}}+n{E}_{\hat{\overset{\sim }{\beta }}}\right)}^{-1}, $$

where ${H}_{\hat{\overset{\sim }{\beta }}}=\sum \limits_{i=1}^N\tilde{x}_{i}^{\prime }{A}_i^{1/2}{R}_i^{-1}\left(\alpha \right){A}_i^{1/2}\tilde{x}_{i}$, ${E}_{\hat{\overset{\sim }{\beta }}}= tr\left({B}^{\prime }B\right)$, and ${M}_{\hat{\overset{\sim }{\beta }}}=\sum \limits_{i=1}^N\tilde{x}_{i}^{\prime }{A}_i^{1/2}{R}_i^{-1}\left(\alpha \right){e}_{\hat{\overset{\sim }{\beta }}}{e}_{\hat{\overset{\sim }{\beta}}}^{\prime }{R}_i^{-1}\left(\alpha \right){A}_i^{1/2}\tilde{x}_{i}$ with ${e}_{\hat{\overset{\sim }{\beta }}}={A}_i^{1/2}\left(\tilde{y}_{i}-{\overset{\sim }{\mu}}_i\right)$. The other indirect way is to calculate it as the sample covariance of ${\overset{\sim }{\boldsymbol{\beta}}}_k$ from permutations. We use this indirect way to reduce computational burden.

For the calculated *p*-values, we implemented two types of multiple testing procedure as we discussed earlier [10]. In short, we applied two approaches: Westfall & Young permutation procedure [32] that effectively considers the correlation of *p*-values, and the Benjamini-Hochberg (BH) step-up procedure [23] that computes *q*-values by False Discovery Rate (FDR) adjustment.

### Simulation study

We conducted a simple simulation study to investigate the performance of PHARAOH-GEE and to compare the proposed method with the existing methods. We first simulated a large pool of rare genetic variants using SimRare [33]. All simulation settings were unchanged except for the 1Kbp of gene length. From the pool, one thousands of replicates were generated, each of those consists of 1000 individuals and 10 pathways. Finally, the phenotypes were simulated from the below model that assumes only the first pathway is causal:

12 $$ g\left({\mu}_{iq}\right)={\eta}_{iq}={\beta}_{1q}\tilde{f}_{i1}={\beta}_{1q}\sum \limits_{t=1}^{H_1}{w}_{1t}{x}_{i1t}={\beta}_{1q}\sum \limits_{t=1}^{H_1}\left({w}_{1t}\sum \limits_{j=1}^{M_{1t}}{\gamma}_{1 tj}{g}_{i1 tj}\right), $$

where *H*_1_ and *M*_1*t*_ denote the number of causal genes in the causal pathway and the number of causal rare variants in the *t*^th^ causal gene, respectively. Note that *M*_1*t*_ was the number of rare variants in the simulated gene varies and was used as an input variable in our simulation study. We set *γ*_1*tj*_ to |log_10_*MAF*_*tj*_|, which represents the effect of the *j*^th^ genetic variant of the *t*^th^ gene. For the simplicity, we generated the phenotypes from the simulated linear predictor *η*_*iq*_, by using it as a binarization threshold from the randomly generated variables from the multivariate normal distribution MVN(0, Σ). For each replicate, all rare variants were collapsed into genes.

### Exome sequencing dataset with clustered phenotypes

In order to illustrate PHARAOH-GEE for investigating associations between multiple pathways and the clustered phenotypes, we analyzed a large-scale WES dataset from a Korean population cohort. Our WES dataset consists of next-generation sequencing data of 1087 individuals’ genomes, using the Illumina HiSeq2000 platform (Illumina, Inc., San Diego, CA), as a part of the T2D-GENES consortium [34]. All individuals of the dataset were originated from a large Korean cohort named the Korean Association REsource (KARE) study [35]. For our analysis, we selected six phenotypes related to the metabolic disease: SBP, DBP, TG, FASTGLU, WAIST and HDL. In our analysis, we considered 995 individuals with complete phenotypes of interest. We then applied two pathway databases Biocarta and KEGG from Molecular Signatures Database [36], which is a curated collection of multiple pathway databases.

## Abbreviations

BH: Benjamini-Hochberg
CV: Cross-validation
DBP: Diastolic blood pressure
FASTGLU: Fasting glucose
FDR: False Discovery Rate
GEE: Generalized estimating equations
GWAS: Genome-wide association studies
HDL: High-density lipoprotein
IDF: International Diabetes Federation
KARE: Korean Association REsource
SBP: Systolic blood pressure
TG: Triglycerides
WAIST: Waist circumference
WES: Whole exome sequencing
